# Modelling and Design of MEMS Piezoresistive Out-of-Plane Shear and Normal Stress Sensors

**DOI:** 10.3390/s18113737

**Published:** 2018-11-02

**Authors:** Yi Zhang, Lin Li

**Affiliations:** 1 Department of Engineering Mechanics, College of Pipeline and Civil Engineering, China University of Petroleum (East China), Qingdao 266580, China; zhangyi@upc.edu.cn; 2 School of Petroleum Engineering, Shandong Provincial Key Laboratory of Oilfield Chemistry, China University of Petroleum (East China), No. 66, Changjiang West Road, Qingdao 266580, China

**Keywords:** MEMS stress sensor, FEM, Out-of-plane shear and normal stress

## Abstract

In this paper, the design of MEMS piezoresistive out-of-plane shear and normal stress sensor is described. To improve the sensor sensitivity, a methodology by the incorporation of stress concentration regions, namely surface trenches in the proximity of sensing elements was explored in detail. The finite element (FE) model, verified by a five-layer analytical model was developed as a tool to model the performance of the sensor and guide the geometric optimization of the surface trenches. Optimum location and dimensions of the surface trenches have been obtained through a comprehensive FE analysis. The microfabrication and packing scheme was introduced to prototype the sensor with optimum geometric characteristics of surface trenches. Signal output from the prototyped sensor was tested and compared with those from FE simulation. Good agreement has been achieved between the simulation and experimental results. Moreover, the results suggest the incorporation of surface trenches can help improve the sensor sensitivity. More specifically, the sum of signal output from the sensor chip with surface trenches are 4.52, 5.06 and 5.72 times higher compared to flat sensor chip for center sensing area, edge sensing areas 1 and 2, respectively.

## 1. Introduction

**Figure 1:**
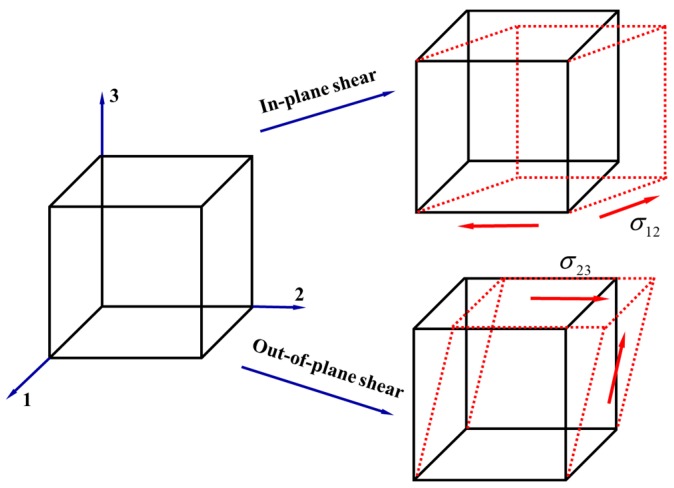
In-plane and out-of-plane shear stress.

Measuring stresses or strains plays an important role in structural health monitoring (SHM), biomechanical engineering, electronic packaging, reliability analysis, etc. As one of the most effective measurement techniques, traditional metal strain gauges have been widely used for decades. Recently, with the development of micro-electrical-mechanical systems (MEMS), piezoresistive strain gauges with higher sensitivity have been proposed for the solution of stress/strain measurement. However, both metal and piezoresistive strain gauges are designed for in-plane stress/strain measurement, as shown in Figure 1. Out-of-plane normal and shear stress sensors are crucial for the development of humanoid robots, biomedical devices such as prosthetic socket system and bladder pressure sensor, microfluidic pump, and so on. As a result, the development of piezoresistive out-of-plane normal and shear stress sensors is urgently required and has attracted wide attention.

A great number of studies report on the development of out-of-plane shear stress sensors. A majority of these shear stress sensors are wall shear stress sensor, which is used to measure shear stress or velocity in flows and gases. A comprehensive literature review about wall shear stress sensors and error analysis related to wall shear stress transducers can be found in [1].

Tactile sensors, widely used for robotic automation and multi-touch applications are another important type of out-of-plane shear stress sensor [2]. In addition, the shear force sensors installed in “Smart Tires” can be used to measure shear force between the tire and road and improve the safety of car driving [3]. Based on the sensing techniques used to transduce shear force or stress into electrical data, out-of-plane shear stress sensor can be classified into the following categories:

### 1.1. Resistive

The transduction method which has received the most attention is to consider the resistance change of conductive material (metal or semiconductor) due to applied shear force or shear stress. Noda et al. [4] developed a shear stress sensor through embedding a piezoresistive cantilever in elastic material. The cantilever standing in elastic material will bend when the elastic material is under deformation. The shear stress components can thus be detected by measuring the resistance change of piezoresistor located at the hinge of the cantilever. In addition to strain gauge or resistor, boss-diaphragm sensor structure is also widely adopted for the development of shear stress sensors. For example, Hwang et al. [5] proposed a polymer-based tactile sensor, which consists of a micromachined polydimethylsiloxane (PDMS) structure and a flexible printed circuit board (FPCB). It is worth mentioning that polymer-based sensors are dependent on the operating voltage [6,7,8,9], which is different from silicon-based sensing solution. A silicon-based shear stress sensor based on diaphragm sensor structure was developed by Wang and Beebe [10,11]. Four ion-implanted resistors are embedded in the diaphragm structure which is similar to a standard diaphragm pressure sensor. Shear forces can be determined based on the change of resistance in these four resistors caused by the deformation of diaphragm. Hsieh [12] designed a micro-shear stress sensor utilizing four-terminal silicon pressure structure and piezoresistiors for an Above-Knee Prosthesis Application. Using liquid metal alloy encapsulated in PDMS substrate as piezoresistive material, and adopting diaphragm sensor structure, a normal and shear force sensor was developed [13,14]. Lemke et al. developed a piezoresistive CMOS out-of-plane shear stress sensor [15], which can measure two out-of-plane shear stress components $\sigma_{xz}$ and $\sigma_{yz}$.

### 1.2. Inductive

Djuric [16] and Damnjanovic [17] proposed a novel inductive displacement sensor which can measure both normal and shear forces. This inductive displacement sensor developed in PCB technology includes two sensor elements, one for vertical displacement detection and the other one for shear displacement measurement. A multiplexed inductive force sensor for simultaneously measuring normal and shear forces on a foot was developed by Du et al. [18]. By monitoring the inductance changes of three planar sensing coils, this inductive sensor is capable of measuring normal force ranging from 0 to 800 N and shear forces ranging from 0 to 130 N in real time. Another inductive tactile sensor, with a sensing range of 0–1.4 N was developed based on a chrome steel ball sensing interface and a deformable polymer layer. The tactile force applied on the chrome steel ball will cause the deformation of polymer, which will lead to the change of distance between the chrome steel ball and the sensing coil [19]. 

### 1.3. Capacitive

It is straightforward that the working principle of capacitor-based shear stress sensor is based on the capacitance change induced by the applied shear force or stress. The capacitive sensing array for robot application presented by Lai et al. [20] includes two sensing electrodes and one floating electrode. Sundara-Rajan et al. [21] designed a shear stress sensor for lower limb prosthetic application. There are three layers in their sensor structure: upper electrode layer encapsulated in PDMS layer, PDMS pillar layer and bottom electrode layer. When there is a shear stress applied on the upper layer, the PDMS pillar would bend along the direction of the force resulting in the capacitance change. Lee et al. [22] reported a flexible polymer normal and shear force sensor with embedded capacitors. 

### 1.4. Fiber Bragg Grating

Fiber Bragg grating (FBG)-based shear stress sensors are designed based on the change of fiber Bragg wavelength or spectral changes caused by the applied shear force. For instance, a novel shear force sensor using FBG as the sensing element consists of layers of carbon composite material and silicon rubber [23]. The deformation of silicon rubber layer induced by the applied shear force would cause the change of grating periodicity and hence the reflected Bragg wavelength. The maximum shear force the presented sensor can measure is approximately 85 N. Candiani et al. [24] proposed an optical fiber shear sensor based on the fusion of microstructured optical fibers and magnetofluidic technologies, the force sensing range of which is 0.42–3.86 N. The change in the relative position of the ferrofluid plug monitored by the Bragg grating would cause the spectral changes, and thus the shear force can be determined from post-evaluation. 

On the other hand, out-of-plane normal stress sensors or the pressure sensors presented in literatures are summarized and compared in Table 1. A variety of sensing principles, such as piezoresistive, capacitive and resonant sensing technology are used in the development of out-of-plane normal stress sensor. However, due to the fragility of diaphragm or membrane structure adopted as the sensor structure, the force sensing range of these out-of-plane normal stress sensors is relatively small. This deficiency is one impediment to the development of a sensing technique for structure health monitoring (SHM) or MEMS packaging where the out-of-plane normal stress can reach as large as 1 GPa.

As a result, research on the development of a MEMS out-of-plane shear and normal stress sensor with large sensing capacity is urgently required. The piezoresistive solid-state MEMS stress sensor shows the potential of being utilized for high load applications. Suhling and Jaeger [25] presented the first piezoresistive stress sensor, which can measure all the six stress components taking temperature effect into consideration. Dual polarity eight sensing elements (four n-type and four p-type) were used in the sensor design but only four stress components are temperature compensated. Later, Gharib and Moussa [26] developed a single polarity 3-D stress sensor which can measure all the six temperature compensated stress components. However, high doping concentration (1 × 10^19^ − 1 × 10^20^ atoms/cm^3^) is adopted in the sensor design in order to reduce the temperature effect on sensor performance. Thus, the piezoresistive coefficients which determine the sensor sensitivity are significantly reduced. In this study, the concept of stress concentration regions (SCRs), generated by the introduction of surface trench is adopted in the sensor design for improving the sensitivity. Furthermore, a 3-D piezoresistive-mechanical coupled finite element model is developed for optimal design of the SCRs, including dimension and position of surface trench. In addition, the microfabrication process for the proposed out-of-plane shear and normal stress sensor is also presented.

## 2. Sensor Design

### 2.1. Piezoresistive Theory

**Figure 2:**
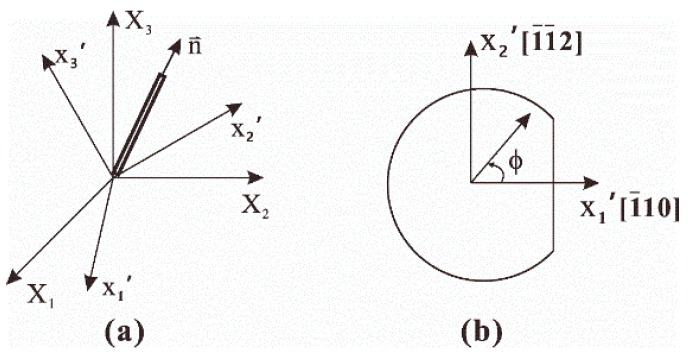
(**a**) Filamentary silicon conductor and (**b**) (1 1 1) silicon wafer with oriented filament.

The piezoresistive behavior of a sensing element on crystalline silicon depends not only on its orientation, but also on the wafer plane on which it is developed. An arbitrary filamentary silicon conductor with respect to the principle silicon crystallographic coordinate system, which is represented as unprimed coordinate, i.e., $X_{1}=\left[100\right],X_{2}=\left[010\right],X_{3}=\left[001\right]$ is shown in Figure 2a. The arbitrary rotated coordinate system is denoted as primed axes, representing the principal crystallographic directions.

The resistance change of a piezoresistive filament due to applied stress or temperature change taking temperature effect into consideration has been given by [25]:

(1) $$\begin{array}{ll} \frac{\Delta R}{R} & =\frac{R\left(\sigma ,T\right)-R\left(0,0\right)}{R\left(0,0\right)}=\pi \times \sigma +\alpha T\\ & =\left(\pi_{1\alpha }^{\prime }\pi_{\alpha }^{\prime }\right){l^{\prime }}^{2}+\left(\pi_{2\alpha }^{\prime }\pi_{\alpha }^{\prime }\right){m^{\prime }}^{2}+\left(\pi_{3\alpha }^{\prime }\pi_{\alpha }^{\prime }\right){n^{\prime }}^{2}\\ & +2\left(\pi_{4\alpha }^{\prime }\pi_{\alpha }^{\prime }\right)l^{\prime }n^{\prime }+2\left(\pi_{5\alpha }^{\prime }\pi_{\alpha }^{\prime }\right)m^{\prime }n^{\prime }+2\left(\pi_{6\alpha }^{\prime }\pi_{\alpha }^{\prime }\right)l^{\prime }m^{\prime }\\ & +(\alpha_{1}T+\alpha_{2}T^{2}+\cdot \cdot \cdot ) \end{array}$$

where 

R(σ,T): Resistance of resistor with applied stress and temperature change;

R(0,0): Resistance of resistor without applied stress and temperature change;

$\pi_{\alpha \beta }^{\prime }:$ Off-axis temperature dependent piezoresistive coefficients;

$\sigma_{\alpha }^{\prime }:$ Stress under the primed coordinate system;

$\alpha_{1},\alpha_{2}:$ First and second order temperature coefficients of resistance (TCR);

$l^{\prime },m^{\prime },n^{\prime }$: Direction cosines of filament orientation with respect to the primed axes $x_{1}{}^{\prime },x_{2}{}^{\prime },x_{3}{}^{\prime }$.

For simplicity, Equation (1) can be reformulated into:

(2) $$\begin{array}{ll} \frac{\Delta R}{R} & =(B_{1}\cos^{2}\phi +B_{2}\sin^{2}\phi )\sigma_{11}^{\prime }\\ & +(B_{1}\sin^{2}\phi +B_{2}\cos^{2}\phi )\sigma_{22}^{\prime }+B_{3}\sigma_{33}^{\prime }\\ & +2\sqrt{2}(B_{2}-B_{3})(\cos^{2}\phi -\sin^{2}\phi )\sigma_{23}^{\prime }\\ & +2\sqrt{2}(B_{2}-B_{3})\sin(2\phi )\sigma_{13}^{\prime }\\ & +(B_{1}-B_{2})\sin(2\phi )\sigma_{12}^{\prime }+\alpha T \end{array}$$

where ϕ is the angle between filamentary conductor and primed coordinate system on (111) silicon wafer as shown in Figure 2b. $B_{1}$, $B_{2}$ and $B_{3}$ are linear combination of piezoresistive coefficients $\pi_{11}$, $\pi_{12}$ and $\pi_{44}$:

(3) $$B_{1}=\frac{\pi_{11}+\pi_{12}+\pi_{44}}{2},B_{2}=\frac{\pi_{11}+5\pi_{12}-\pi_{44}}{6},B_{3}=\frac{\pi_{11}+2\pi_{12}-\pi_{44}}{3}\;$$

### 2.2. Sensor Description

**Figure 3:**
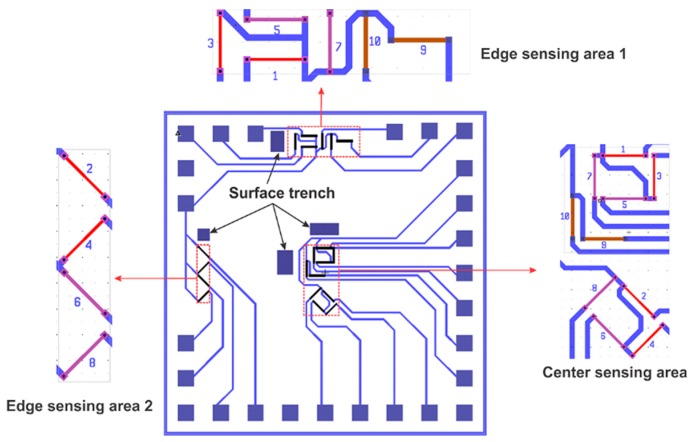
Chip layout out-of-plane shear stress and normal stress sensor developed on (111) wafer.

The proposed out-of-plane shear and normal stress sensor is developed from p-type silicon wafer using single polarity (n-type) diffused sensing elements. As shown in Figure 3, there are three sensing areas on the silicon chip, one center sensing area designed for out-of-plane normal stress measurement and edge sensing areas 1 and 2 for the measurement of out-of-plane shear stress $\sigma_{13}$ and $\sigma_{23}$, respectively. 

There are ten sensing elements in the center sensing area while four and six sensing elements are located in the edge sensing area1 and edge sensing area 2, respectively. Based on their doping concentration, these ten sensing elements can be divided into three groups: group a (sensing element 1–4), group b (sensing element 5–8) and group c (sensing element 9–10). By defining different angles ϕ and coefficients $B_{i}$ in Equation (2) ten linear equations can be obtained:

(4) $$\begin{array}{l} \frac{\Delta R_{1}}{R_{1}}=B_{1}^{a}\sigma_{11}^{\prime }+B_{2}^{a}\sigma_{22}^{\prime }+B_{3}^{a}\sigma_{33}^{\prime }+2\sqrt{2}\left(B_{2}^{a}-B_{3}^{a}\right)\sigma_{23}^{\prime }+\alpha^{a}T\\ \frac{\Delta R_{2}}{R_{2}}=\frac{B_{1}^{a}+B_{2}^{a}}{2}\sigma_{11}^{\prime }+\frac{B_{1}^{a}+B_{2}^{a}}{2}\sigma_{22}^{\prime }+B_{3}^{a}\sigma_{33}^{\prime }\\ \;+2\sqrt{2}\left(B_{2}^{a}-B_{3}^{a}\right)\sigma_{13}^{\prime }+\left(B_{1}^{a}-B_{2}^{a}\right)\sigma_{12}^{\prime }+\alpha^{a}T\\ \frac{\Delta R_{3}}{R_{3}}=B_{2}^{a}\sigma_{11}^{\prime }+B_{1}^{a}\sigma_{22}^{\prime }+B_{3}^{a}\sigma_{33}^{\prime }-2\sqrt{2}\left(B_{2}^{a}-B_{3}^{a}\right)\sigma_{33}^{\prime }+\alpha^{a}T\\ \frac{\Delta R_{4}}{R_{4}}=\frac{B_{1}^{a}+B_{2}^{a}}{2}\sigma_{11}^{\prime }+\frac{B_{1}^{a}+B_{2}^{a}}{2}\sigma_{22}^{\prime }+B_{3}^{a}\sigma_{33}^{\prime }\\ \;-2\sqrt{2}\left(B_{2}^{a}-B_{3}^{a}\right)\sigma_{13}^{\prime }-\left(B_{1}^{a}-B_{2}^{a}\right)\sigma_{12}^{\prime }+\alpha^{a}T\\ \frac{\Delta R_{5}}{R_{5}}=B_{1}^{b}\sigma_{11}^{\prime }+B_{2}^{b}\sigma_{22}^{\prime }+B_{3}^{b}\sigma_{33}^{\prime }+2\sqrt{2}\left(B_{2}^{b}-B_{3}^{b}\right)\sigma_{23}^{\prime }+\alpha^{b}T\\ \frac{\Delta R_{6}}{R_{6}}=\frac{B_{1}^{b}+B_{2}^{b}}{2}\sigma_{11}^{\prime }+\frac{B_{1}^{b}+B_{2}^{b}}{2}\sigma_{22}^{\prime }+B_{3}^{b}\sigma_{33}^{\prime }\\ \;+2\sqrt{2}\left(B_{2}^{b}-B_{3}^{b}\right)\sigma_{13}^{\prime }+\left(B_{1}^{b}-B_{2}^{b}\right)\sigma_{12}^{\prime }+\alpha^{b}T\\ \frac{\Delta R_{7}}{R_{7}}=B_{2}^{b}\sigma_{11}^{\prime }+B_{1}^{b}\sigma_{22}^{\prime }+B_{3}^{b}\sigma_{33}^{\prime }-2\sqrt{2}\left(B_{2}^{b}-B_{3}^{b}\right)\sigma_{23}^{\prime }+\alpha^{b}T\\ \frac{\Delta R_{8}}{R_{8}}=\frac{B_{1}^{b}+B_{2}^{b}}{2}\sigma_{11}^{\prime }+\frac{B_{1}^{b}+B_{2}^{b}}{2}\sigma_{22}^{\prime }+B_{3}^{b}\sigma_{33}^{\prime }\\ \;-2\sqrt{2}\left(B_{2}^{b}-B_{3}^{b}\right)\sigma_{13}^{\prime }-\left(B_{1}^{b}-B_{2}^{b}\right)\sigma_{12}^{\prime }+\alpha^{b}T\\ \frac{\Delta R_{9}}{R_{9}}=B_{1}^{c}\sigma_{11}^{\prime }+B_{2}^{c}\sigma_{22}^{\prime }+B_{3}^{c}\sigma_{33}^{\prime }+2\sqrt{2}\left(B_{2}^{c}-B_{3}^{c}\right)\sigma_{23}^{\prime }+\alpha^{c}T\\ \frac{\Delta R_{10}}{R_{10}}=B_{2}^{c}\sigma_{11}^{\prime }+B_{1}^{c}\sigma_{22}^{\prime }+B_{3}^{c}\sigma_{33}^{\prime }-2\sqrt{2}\left(B_{2}^{c}-B_{3}^{c}\right)\sigma_{23}^{\prime }+\alpha^{c}T \end{array}$$

where superscripts a, b and c indicate three different groups of sensing elements. All the six stress components, including out-of-plane shear and normal stresses $\sigma_{13}$, $\sigma_{23}$ and $\sigma_{33}$ can be solved from Equation (3):

(5) $$\begin{array}{l} \sigma_{11}^{\prime }=\frac{1}{2D_{2}}[(B_{3}{}^{c}\alpha^{b}-B_{3}{}^{b}\alpha^{c})\left(\frac{\Delta R_{1}}{R_{1}}+\frac{\Delta R_{3}}{R_{3}}\right)+(B_{3}{}^{a}\alpha^{c}-B_{3}{}^{c}\alpha^{a})\left(\frac{\Delta R_{5}}{R_{5}}+\frac{\Delta R_{7}}{R_{7}}\right)\\ \;+(B_{3}{}^{b}\alpha^{a}-B_{3}{}^{a}\alpha^{b})\left(\frac{\Delta R_{9}}{R_{9}}+\frac{\Delta R_{10}}{R_{10}}\right)]\\ \;+\frac{1}{2D_{1}}\left[(B_{2}{}^{b}-B_{3}{}^{b})\left(\frac{\Delta R_{1}}{R_{1}}-\frac{\Delta R_{3}}{R_{3}}\right)+(B_{2}{}^{a}-B_{3}{}^{a})\left(\frac{\Delta R_{5}}{R_{5}}-\frac{\Delta R_{7}}{R_{7}}\right)\right]\\ \sigma_{22}^{\prime }=\frac{1}{2D_{2}}[(B_{3}{}^{c}\alpha^{b}-B_{3}{}^{b}\alpha^{c})\left(\frac{\Delta R_{1}}{R_{1}}+\frac{\Delta R_{3}}{R_{3}}\right)+(B_{3}{}^{a}\alpha^{c}-B_{3}{}^{c}\alpha^{a})\left(\frac{\Delta R_{5}}{R_{5}}+\frac{\Delta R_{7}}{R_{7}}\right)\\ \;+(B_{3}{}^{b}\alpha^{a}-B_{3}{}^{a}\alpha^{b})\left(\frac{\Delta R_{9}}{R_{9}}+\frac{\Delta R_{10}}{R_{10}}\right)]\\ \;-\frac{1}{2D_{1}}\left[(B_{2}{}^{b}-B_{3}{}^{b})\left(\frac{\Delta R_{1}}{R_{1}}-\frac{\Delta R_{3}}{R_{3}}\right)-(B_{2}{}^{a}-B_{3}{}^{a})\left(\frac{\Delta R_{5}}{R_{5}}-\frac{\Delta R_{7}}{R_{7}}\right)\right]\\ \sigma_{33}^{\prime }=\frac{1}{2D_{2}}[\left(\left(B_{1}{}^{b}+B_{2}{}^{b}\right)\alpha^{c}-\left(B_{1}{}^{c}+B_{2}{}^{c}\right)\alpha^{b}\right)\left(\frac{\Delta R_{1}}{R_{1}}+\frac{\Delta R_{3}}{R_{3}}\right)\\ \;+\left(\left(B_{1}{}^{c}+B_{2}{}^{c}\right)\alpha^{a}-\left(B_{1}{}^{a}+B_{2}{}^{a}\right)\alpha^{c}\right)\left(\frac{\Delta R_{5}}{R_{5}}+\frac{\Delta R_{7}}{R_{7}}\right)\\ \;+\left(\left(B_{1}{}^{a}+B_{2}{}^{a}\right)\alpha^{b}-\left(B_{1}{}^{b}+B_{2}{}^{b}\right)\alpha^{a}\right)\left(\frac{\Delta R_{9}}{R_{9}}+\frac{\Delta R_{10}}{R_{10}}\right)]\\ \sigma_{12}^{\prime }=\frac{1}{D_{1}}[-\frac{(B_{2}{}^{b}-B_{3}{}^{b})}{2}\left(\frac{\Delta R_{2}}{R_{2}}-\frac{\Delta R_{4}}{R_{4}}\right)-\frac{(B_{2}{}^{a}-B_{3}{}^{a})}{2}\left(\frac{\Delta R_{6}}{R_{6}}-\frac{\Delta R_{8}}{R_{8}}\right)]\\ \sigma_{13}^{\prime }=\frac{1}{D_{1}}[-\frac{(B_{1}{}^{b}-B_{2}{}^{b})}{4\sqrt{2}}\left(\frac{\Delta R_{2}}{R_{2}}-\frac{\Delta R_{4}}{R_{4}}\right)+\frac{(B_{1}{}^{a}-B_{2}{}^{a})}{4\sqrt{2}}\left(\frac{\Delta R_{6}}{R_{6}}-\frac{\Delta R_{8}}{R_{8}}\right)]\\ \sigma_{23}^{\prime }=\frac{1}{D_{1}}[-\frac{(B_{1}{}^{b}-B_{2}{}^{b})}{4\sqrt{2}}\left(\frac{\Delta R_{1}}{R_{1}}-\frac{\Delta R_{3}}{R_{3}}\right)+\frac{(B_{1}{}^{a}-B_{2}{}^{a})}{4\sqrt{2}}\left(\frac{\Delta R_{5}}{R_{5}}-\frac{\Delta R_{7}}{R_{7}}\right)] \end{array}$$

Two edge sensing areas are located at the edge of the chip because out-of-plane shear stress reaches its maximum value near the edge of sensor chip while it remains nearly zero at the center of the chip according to shear lag theory [40,41]. As a result, the sensor sensitivity can be improved significantly. Moreover, the edge sensing area can monitor the most dangerous spot of bonding layers, where maximum shear stress occurs. What is more important, SCRs, namely surface trenches are created near the sensing areas which also help improve the sensitivity of the sensors.

**Figure 4:**
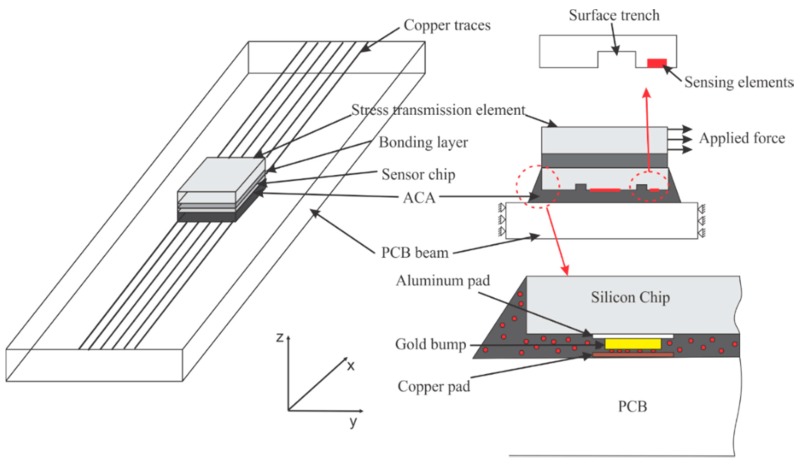
Schematic description of out-of-plane shear and normal stress sensor system.

To verify the capacity of the proposed out-of-plane stress sensor in extraction of the out-of-plane shear and normal stresses, the sensor chip is flip-chipped on a printed circuit board (PCB) beam using anisotropic conductive adhesive (ACA) as shown in Figure 4. The copper pads on the sensor chip are connected with those on the PCB through Gold bump. The shear stress is transmitted to the sensor chip by applying force to the stress transmission element bonded to the bottom of the chip.

## 3. Finite Element Model

**Figure 5:**
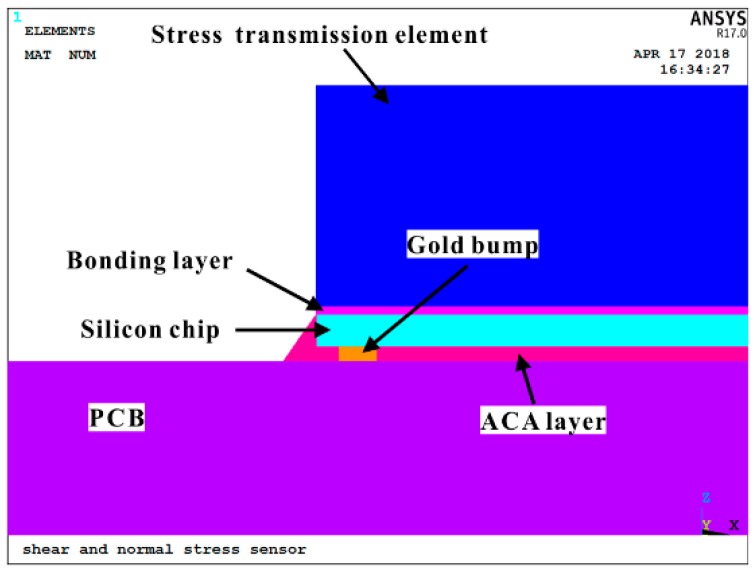
Schematic description of the FEM, including stress transmission element, bonding layer, silicon chip, ACA and PCB.

**Figure 6:**
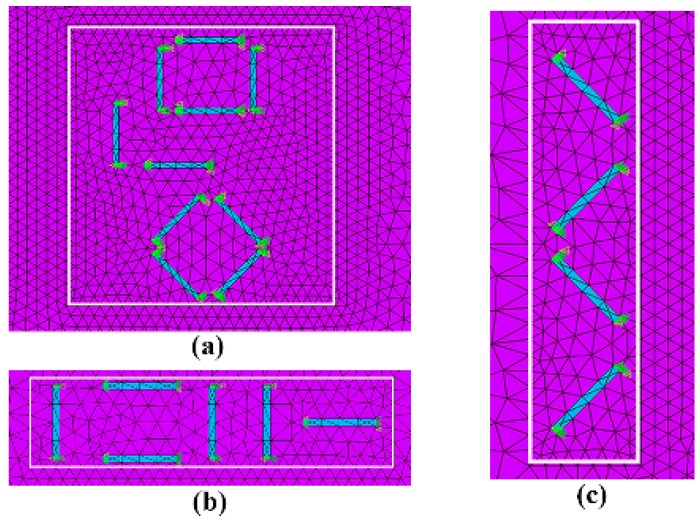
Mesh of the (**a**) center sensing area, (**b**) edge sensing area 1 and (**c**) edge sensing area 2.

A piezoresistive-mechanical coupled finite element model was developed using commercial finite element analysis (FEA) software, ANSYS Multiphysics. As shown in Figure 5, the finite element model (FEM) comprises of five layers, including PCB layer, ACA layer, silicon chip (sensor), bonding layer and stress transmission layer, which is the same as the out-of-plane shear stress and normal sensor system presented in Figure 4. It is deserved to mention that the aluminum pads at the silicon chip are connected with the PCB through gold bumps embedded in the ACA and the cross section of spew fillet caused by flowing of adhesive is modeled as triangular. The gold bumps are simplified as cylinders with diameter of 350 µm and thickness of 70 µm. Sensing elements, namely piezoresistors are modeled as bricks with length, width and height measuring 200 µm, 20 µm and 5 µm, respectively. Mesh of piezoresistors located in three sensing areas is presented in Figure 6. Stress transmission element, PCB, bonding layer and ACA are considered as isotropic material while silicon chip is considered anisotropic. Material properties and geometries of each component are shown in Table 2. Since the FEM is developed to solve piezoresistive-mechanical coupled problem, SOLID187 10-noded tetrahedral elements are used for structural components and SOLID226 10-noded structural-PR coupled tetrahedral elements are used for piezoresistors. 

**Figure 7:**
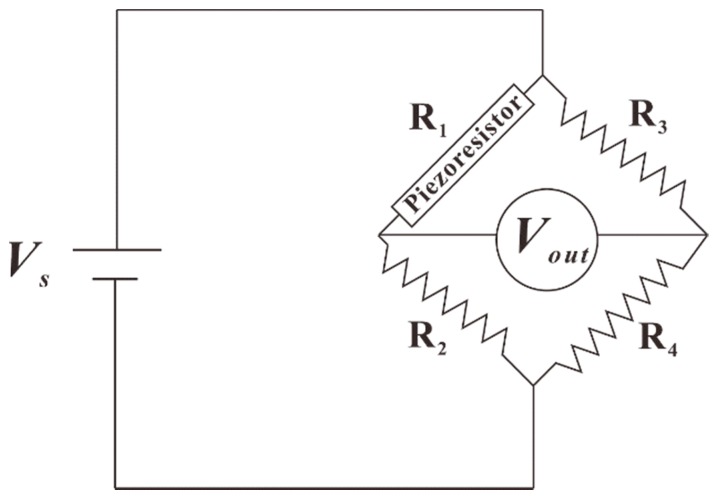
Wheatstone bridge configuration.

Each piezoresistior on the silicon chip is connected in a Wheatstone bridge, as shown in Figure 7 with three CIRCU124 resistor elements measuring the same resistance. The resistance change of each resistor is calculated from the voltage output of the Wheatstone bridge as shown below:

(6) $$\frac{\Delta R}{R}=\frac{4\Delta V/V_{s}}{1-2\Delta V/V_{s}}\;$$

where $V_{s}$ is the voltage applied to the Wheatstone bridge, which is 5 V and ΔV is the voltage change after the force is applied to the stress transmission element. 

## 4. Analytical Model for Five-Layer Structure

**Figure 8:**
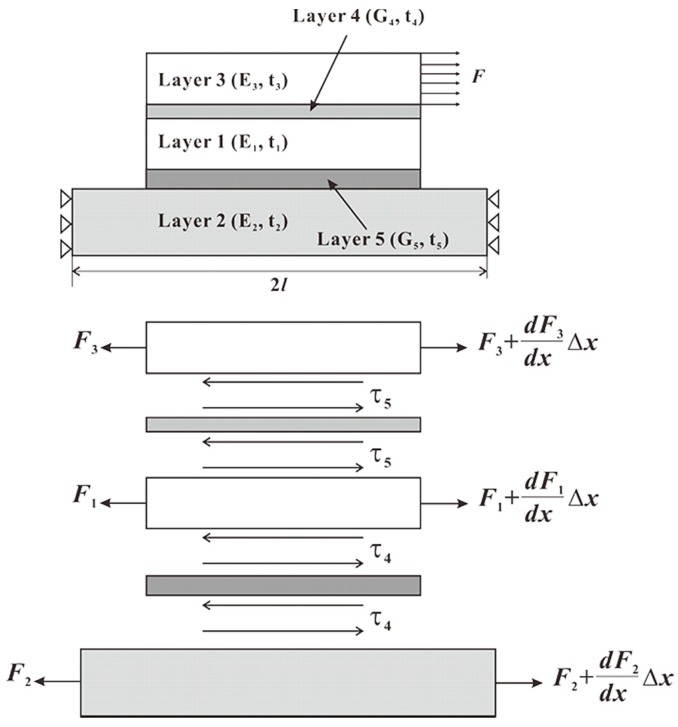
Force equilibrium diagram for five-layer analytical model.

To verify the feasibility of the FEM, out-of-plane shear stresses were derived from a five-layer analytical model and compared with those from the FEM. As shown in Figure 8, the following equations can be derived based on the equilibrium of forces in x direction:

(7) $$\begin{array}{l} \frac{dF_{1}}{dx}+\tau_{4}-\tau_{5}=0\\ \frac{dF_{2}}{dx}-\tau_{4}=0\\ \frac{dF_{3}}{dx}+\tau_{5}=0 \end{array}$$

The stress-strain relationships for layer 1, layer 2 and layer 3 can be described as:

(8) $$\begin{array}{l} \frac{du_{1}}{dx}=\frac{F_{1}}{E_{1}t_{1}}\\ \frac{du_{2}}{dx}=\frac{F_{2}}{E_{2}t_{2}}\\ \frac{du_{3}}{dx}=\frac{F_{3}}{E_{3}t_{3}} \end{array}$$

where $E_{i}$ and $t_{i}$ are the elastic modulus and thickness for the *i-*th layer. And the stress-strain relationships for bonding layers can be written as:

(9) $$\begin{array}{l} \frac{\tau_{4}}{G_{4}}=\frac{u_{1}-u_{2}}{t_{4}}\\ \frac{\tau_{5}}{G_{5}}=\frac{u_{3}-u_{1}}{t_{4}} \end{array}$$

where $G_{i}$ is the shear modulus for the *i-*th layer. The solutions for the above equations can be solved easily and the general solutions for shear stress can be expressed as:

(10) $$\tau_{4}=\tau_{5}=Ae^{\beta x}+Be^{-\beta x}\;$$

where $\beta^{2}=\left(\frac{\frac{1}{E_{2}t_{2}t_{4}}+\frac{1}{E_{3}t_{3}t_{5}}}{\frac{1}{G_{4}}+\frac{1}{G_{4}}}\right)$.

Taking boundary conditions into consideration we can obtain the expression for shear stress:

(11) $$\tau_{4}=\tau_{5}=\frac{F\beta }{2w(e^{\beta l}-e^{-\beta l})}\left(e^{\beta x}+Be^{-\beta x}\right)\;$$

where w is width of layer 5.

## 5. Results

### 5.1. FEM vs. Analytical Model

**Figure 9:**
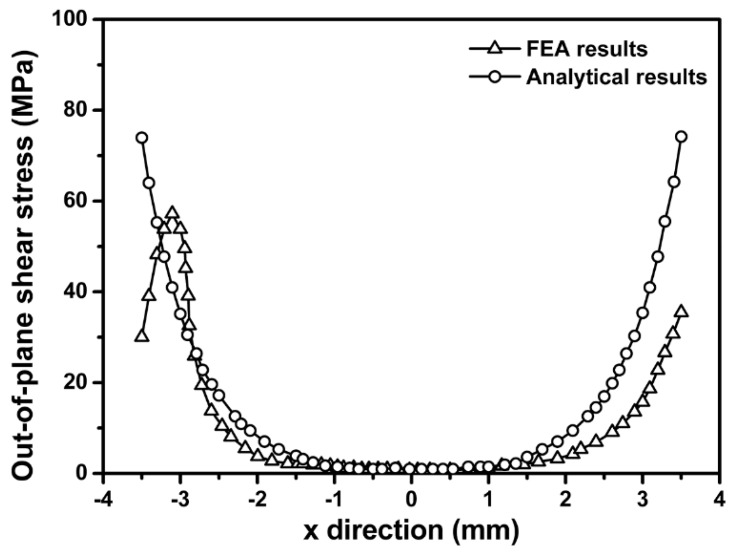
Out-of-plane shear stress distribution on silicon chip derived from analytical model and FEM.

Out-of-plane shear stress $\sigma_{xz}$ along x direction is determined using both FEM and analytical model. Based on classical analysis on single-lap joints presented by Volkersen [40] and Goland and Reissner [41] the maximum out-of-plane shear stress $\sigma_{xz}$ occurs near the edge of bonding layer while is close to zero in the middle area, which is in a good agreement with our results. Furthermore, Figure 9 suggests the results from the proposed FEM are close to those calculated based on the analytical model. The discrepancy between the FEA and analytical results at the region where *x* ≤−3 mm and *x* ≥3 mm is due to the existence of the Gold bump in the FEM, which is not taken into consideration in the analytical model.

### 5.2. Effect of Surface Trench on Signal Output

**Figure 10:**
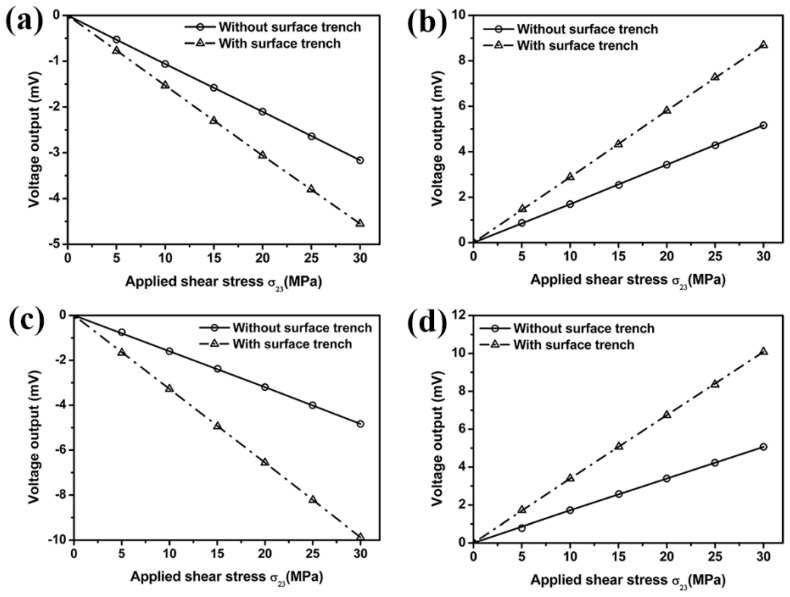
Effect of surface trench on the voltage output from resistors 1 (**a**), 3 (**b**), 5 (**c**) and 7 (**d**) located in edge sensing area 1.

**Figure 11:**
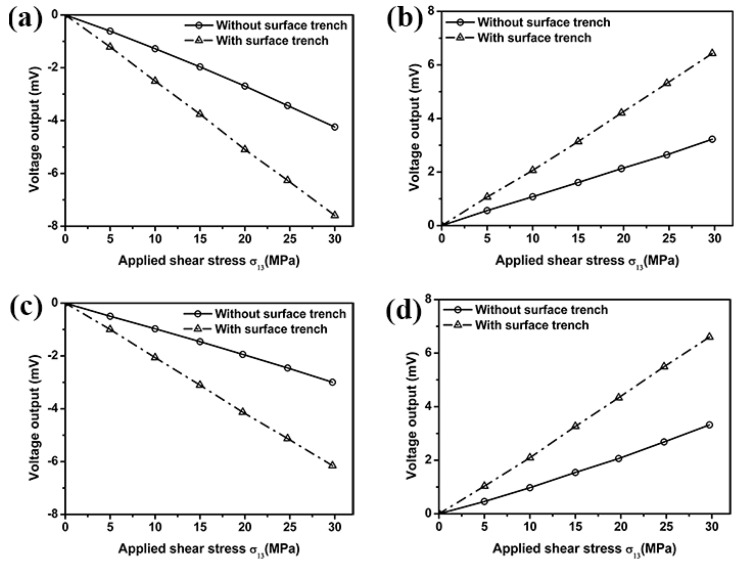
Effect of surface trench on the voltage output from resistors 2 (**a**), 4 (**b**), 6 (**c**) and 8 (**d**) located in edge sensing area 2.

The surface trenches are introduced in our sensor design through Reactive Ion Etching (RIE) technique to generate a stress concentration area near the sensing area. The influence of surface trench on the signal output is investigated by comparing results from a silicon chip without surface trench, namely flat chip and a silicon chip with surface trenches using the FEM. Effects of the surface trench on the sensitivity of out-of-plane shear stress sensor are studied by applying a shear force to the stress transmission element. Voltage output from resistors 1, 3, 5 and 7 in edge sensing area 1, and resistors 2, 4, 6 and 8 in edge sensing area 2 located on a flat silicon chip is determined from the FEM and compared with that from silicon chip with surface trenches. The results in Figure 10 and Figure 11 suggest voltage output in edge sensing areas 1 and 2, respectively, has been increased due to the existence of surface trenches. Accordingly, the sensitivity of the proposed out-of-plane shear stress sensor could be improved significantly.

**Figure 12:**
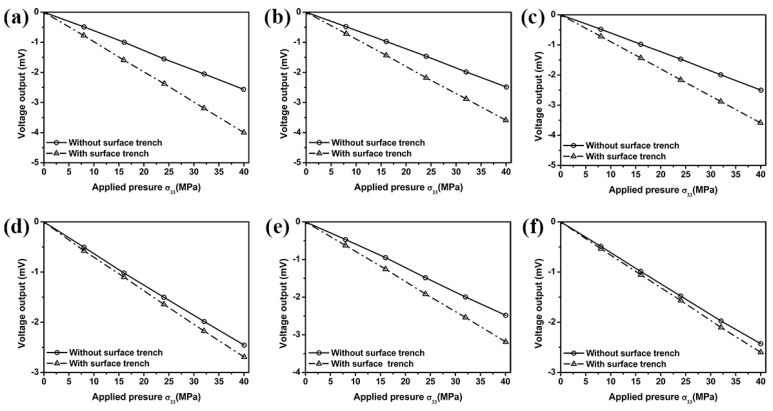
Effect of surface trench on the voltage output of resistors 1 (**a**), 3 (**b**), 5 (**c**), 7 (**d**), 9 (**e**) and 10 (**f**) located in the center sensing area.

In order to evaluate the effect of the surface trench on the sensitivity of out-of-plane normal stress sensor, the voltage output of resistors 1, 3, 5, 7, 9 and 10 from the center sensing area are determined from the FEM when the out-of-plane normal force (pressure) is applied on the top surface of the stress transmission element. The results in Figure 12 show that the existence of surface trenches near the sensing elements help increase the voltage output, thus improve the sensor sensitivity.

### 5.3. Geometric Optimization of Surface Features

In view that the signal output and sensor sensitivity can be improved by introducing surface trenches in the vicinity of sensing areas, we aim to find the optimum location and dimensions of the surface features using the FEM developed in Section 3. Location of surface trench is defined as the distance between the middle of surface trench and sensing element. Dimensions of surface trenches are described as length, width and depth. Since the width of the surface trench is dependent on the location of surface trench, thus considered a constant value of 150 μm. To evaluate the effect of location and dimensions of surface trenches on the sensitivity of out-of-plane shear and normal stress sensors, the percentage signal change is defined as the increase or decrease in each piezoresistor output signal compared to flat sensor chip divided by the output of flat sensor. Therefore, percentage sensitivity change is proportional to percentage signal change. In this study, since center sensing area, edge sensing areas 1 and 2 are composed of more than one piezoresistors, sum of signal change from all the piezoresistors in each sensing area is used to describe the sensor sensitivity of measuring out-of-plane shear and normal stress. 

#### 5.3.1. Out-of-Plane Shear Stress Sensor

For out-of-plane shear stress sensor, $S_{1}$ and $S_{2}$ represent the sum of percentage voltage change from resistors 1, 3, 5 and 7 located in edge sensing area 1, and from resistors 2, 4, 6 and 8 located in edge sensing area 2 respectively:

(12) $$\begin{array}{l} S_{1}=\frac{\Delta V_{1}}{V_{1}}+\frac{\Delta V_{3}}{V_{3}}+\frac{\Delta V_{5}}{V_{5}}+\frac{\Delta V_{7}}{V_{7}}\\ S_{2}=\frac{\Delta V_{2}}{V_{2}}+\frac{\Delta V_{4}}{V_{4}}+\frac{\Delta V_{6}}{V_{6}}+\frac{\Delta V_{8}}{V_{8}} \end{array}$$

where $S_{1}$ and $S_{2}$ are the sum of percentage signal output used to defined out-of-plane shear stress sensor sensitivity, $\Delta V_{i}\left(i=1,\;2,\;3\;\cdots \;8\right)$ are the difference between the output voltage from piezoresistors on flat sensor chip and sensor chip with surface trenches respectively, and $V_{i}\left(i=1,\;2,\;3\;\cdots \;8\right)$ are the signal outputs of piezoresistors located in flat sensor chip.

**Figure 13:**
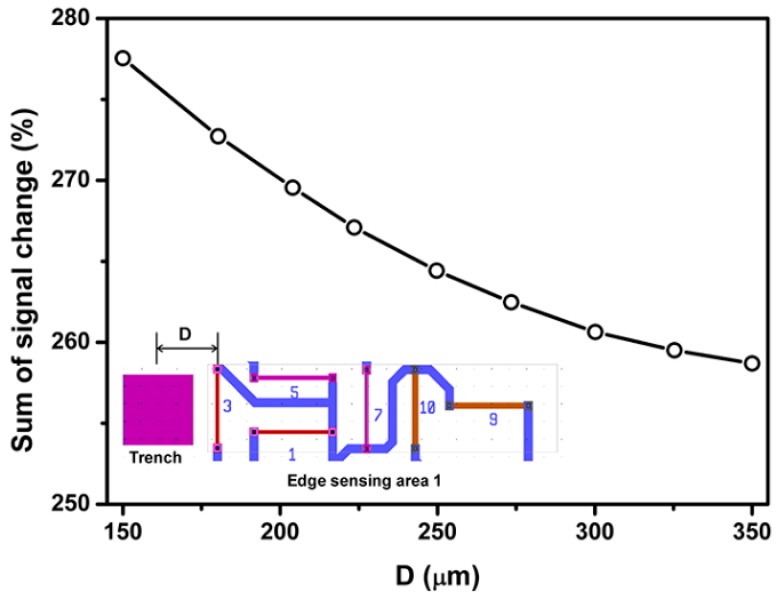
Effect of SCA locations (defined as the distance between surface trench and sensing area, D) on the percentage signal output.

Locations of surface trench in edge sensing areas 1 and 2 are defined as the distance between surface trench and piezoresistors 3 and 2, respectively. Results in Figure 13 show that sum of percentage signal output decreases as the increase of distance between surface trenches and piezoresistor 3. Therefore, the surface trench and piezoresistor 3 should stay as close together as possible. However, due to the microfracation resolution limit the minimum distance between the surface trench and piezoresistor 3 was chosen as the sum of half width of the surface trench (70 µm) and the microfacrication resolution (15 µm), which is 85 µm. It should be noted that the principle for selecting the D value is to maximize the signal output based on the relationship between signal output and D obtained from FE simulation.

**Figure 14:**
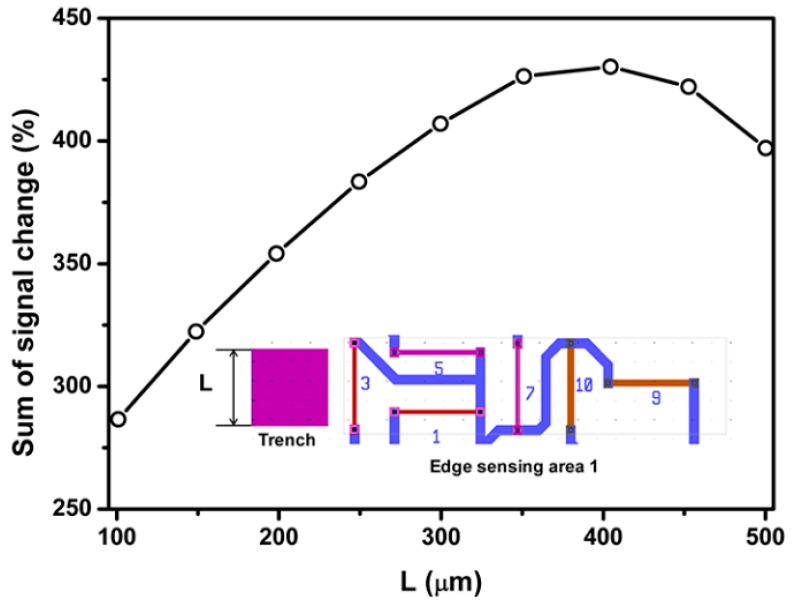
Effect of SCA depth on the percentage signal output sensing area 1.

**Figure 15:**
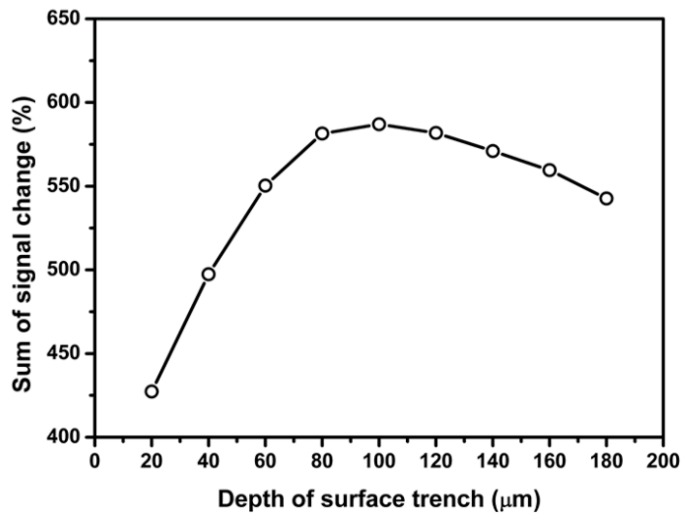
Effect of SCA depth on the percentage signal output sensing area 1.

Figure 14 and Figure 15 present the dependence of sum of percentage signal change on the length and depth of the surface trench, respectively. The results in Figure 14 and Figure 15 show that sum of percentage signal output increases first then decrease with increasing the length and depth of surface trench, respectively. It can be found that the maximum signal output can be attained by choosing the length or depth of surface trench as 400 and 100 µm, respectively. 

**Figure 16:**
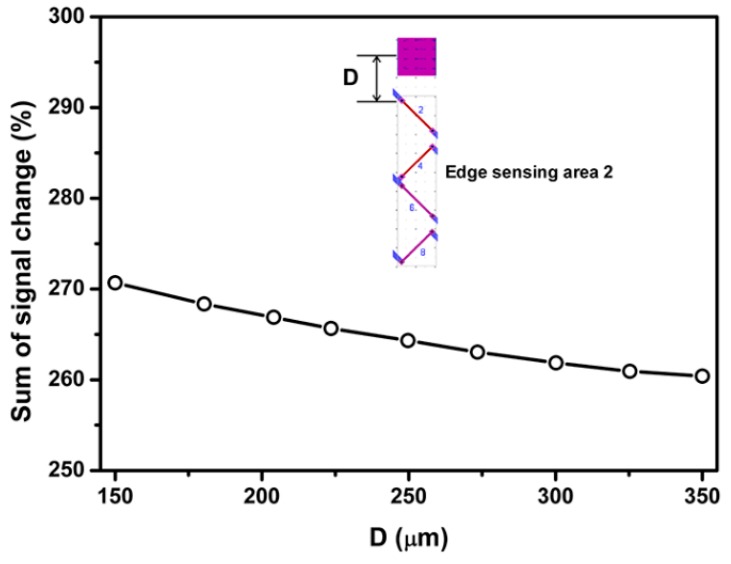
Effect of SCA depth on the percentage signal output sensing area 2.

**Figure 17:**
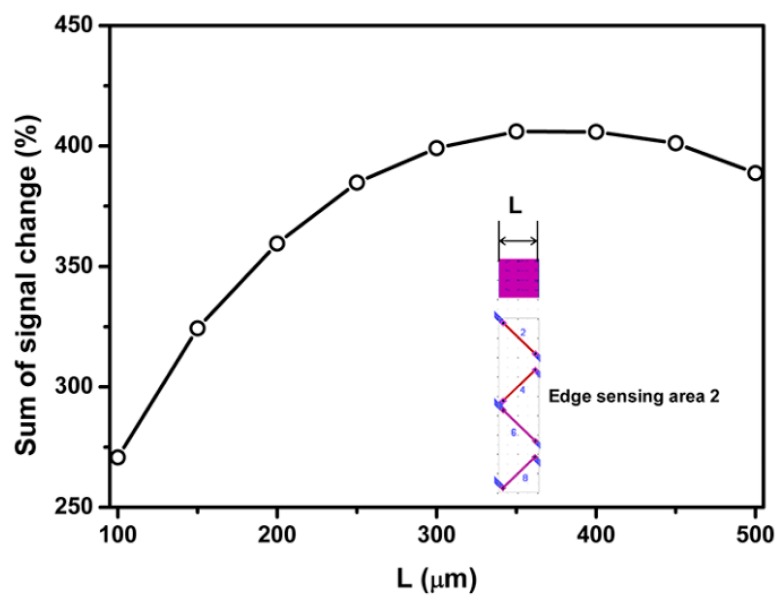
Effect of SCA depth on the percentage signal output sensing area 2.

**Figure 18:**
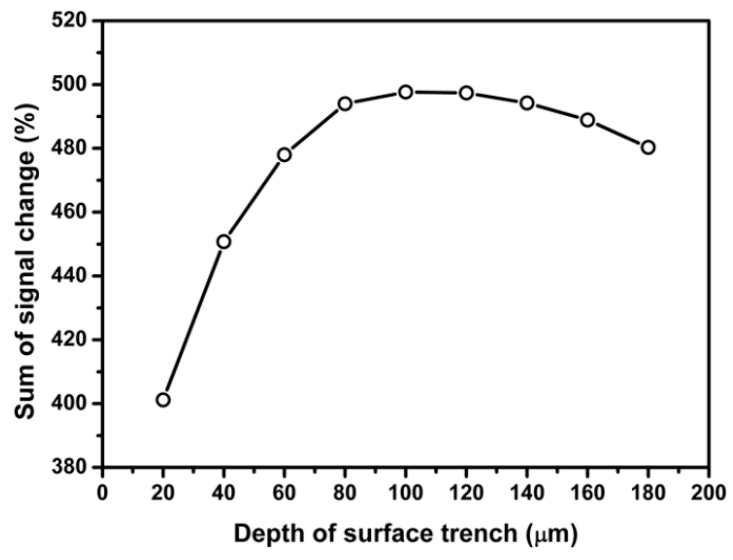
Effect of SCA depth on the percentage signal output sensing area 2.

Regarding edge sensing area 2, sum of percentage signal output as a function of distance between surface trench and piezoresistor 2, length and depth of surface trench is presented in Figure 16, Figure 17 and Figure 18, respectively. 

The trend shown in Figure 16, which is similar to edge sensing area 1, is an increase in the sum of percentage signal output as the distance between surface trenches and piezoresistor 2 decreases. As a result, the distance between the surface trench and piezoresistor 2 was chosen as 85 µm. 

As shown in Figure 17 and Figure 18, the first increase then decrease of the sum of percentage signal output with length and depth of surface trench is also similar to the phenomenon observed for edge sensing area 1. It can be found that the length or depth of surface trench of 350 and 100 µm, respectively provides the maximum sum of percentage signal output. 

#### 5.3.2. Out-of-Plane Normal Stress Sensor

**Figure 19:**
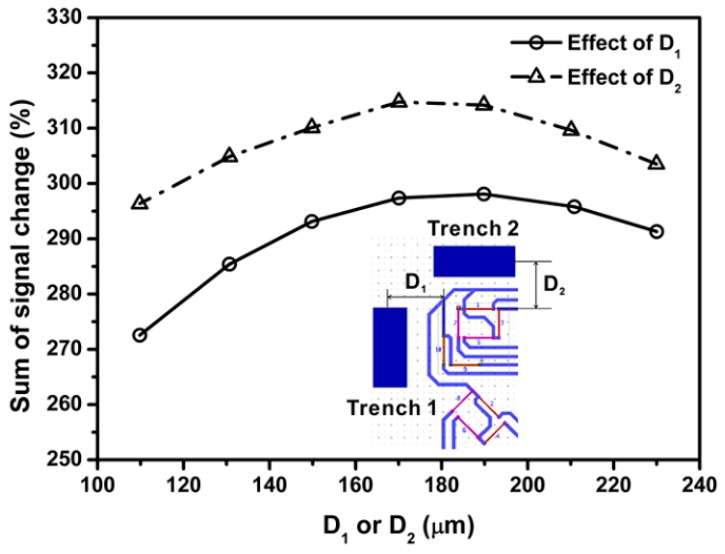
Effect SCA locations (expressed as D1 and D2, which are distances between stress concentration area and sensing elements) on the percentage signal output.

**Figure 20:**
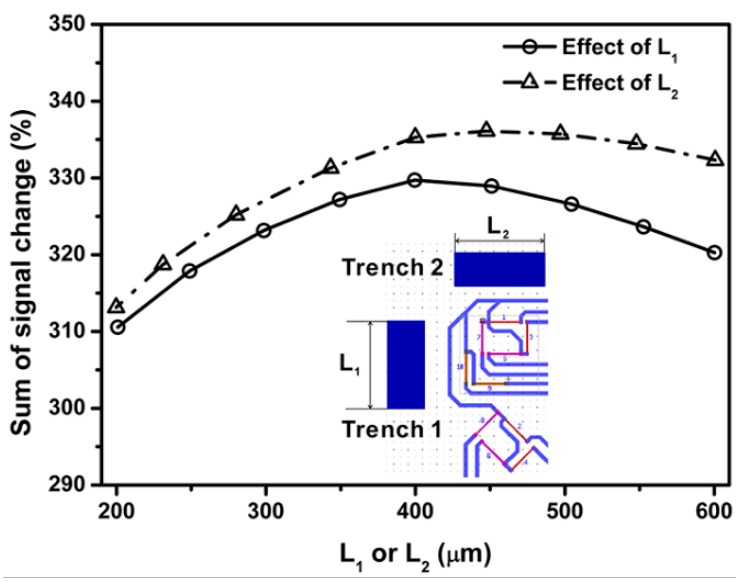
Effect of stress concentration area (SCA) length on the percentage signal output.

**Figure 21:**
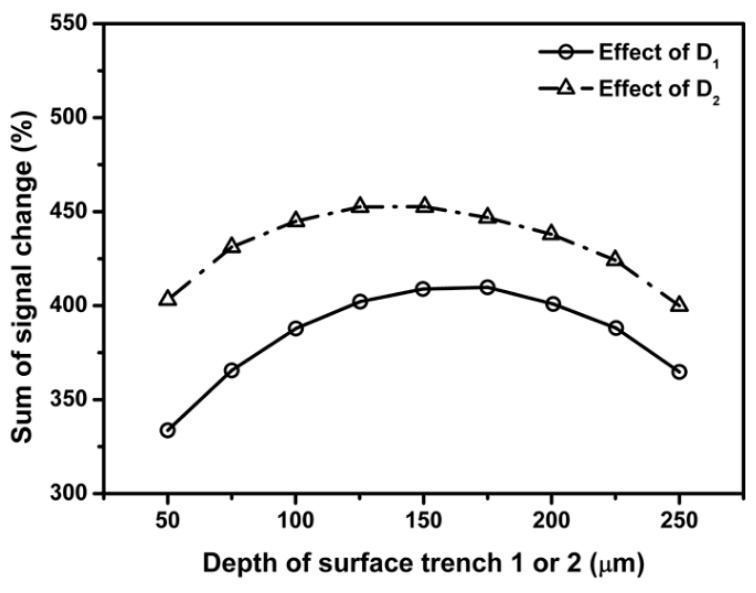
Effect of stress concentration area (SCA) on the percentage signal output.

For out-of-plane normal stress sensor, $S_{3}$ is the sum of percentage voltage change from resistors 1, 3, 5, 7, 9 and 10 located in the center sensing area:

(13) $$S_{3}=\frac{\Delta V_{1}}{V_{1}}+\frac{\Delta V_{3}}{V_{3}}+\frac{\Delta V_{5}}{V_{5}}+\frac{\Delta V_{7}}{V_{7}}+\frac{\Delta V_{9}}{V_{9}}+\frac{\Delta V_{10}}{V_{10}}\;$$

where $\Delta V_{1}$, $\Delta V_{3}$, $\Delta V_{5}$, $\Delta V_{7}$, $\Delta V_{9}$ and $\Delta V_{10}$ are the difference between the output voltage from flat sensor chip and sensor chip with surface trenches, respectively, $V_{1}$, $V_{3}$, $V_{5}$, $V_{7}$, $V_{9}$ and $V_{10}$ are the signal outputs of piezoresistors located in flat sensor chip. Since there are two surface trenches in the center sensing area, the distance between trench 1 and resistor 10 is denoted as $D_{1}$ and the distance between trench 2 and resistor 1 is denoted as $D_{2}$. Figure 19, Figure 20 and Figure 21 present the dependence of sum of percentage signal change on the distance between surface trench, length and depth of surface trench, respectively. The results in Figure 19 show that sum of percentage signal output increases as the distance between surface trenches and piezoresistors. Then the sum of percentage signal change decreases after a specific distance of 190 µm for $D_{1}$ and 180 µm for $D_{2}$, respectively. 

Results in Figure 20 show an increase of the sum of percentage signal change up to a maximum when the lengths of surface trenches 1 and 2 reaches 380 and 400 µm, respectively. Effects of depth of surface trench on the sum of percentage signal change for center sensing area are summarized in Figure 20 and the maximum output signal can be obtained by selecting the depth of trenches 1 and 2 as 175 and 125 µm, respectively.

## 6. Microfabrication Process Flow

**Figure 22:**
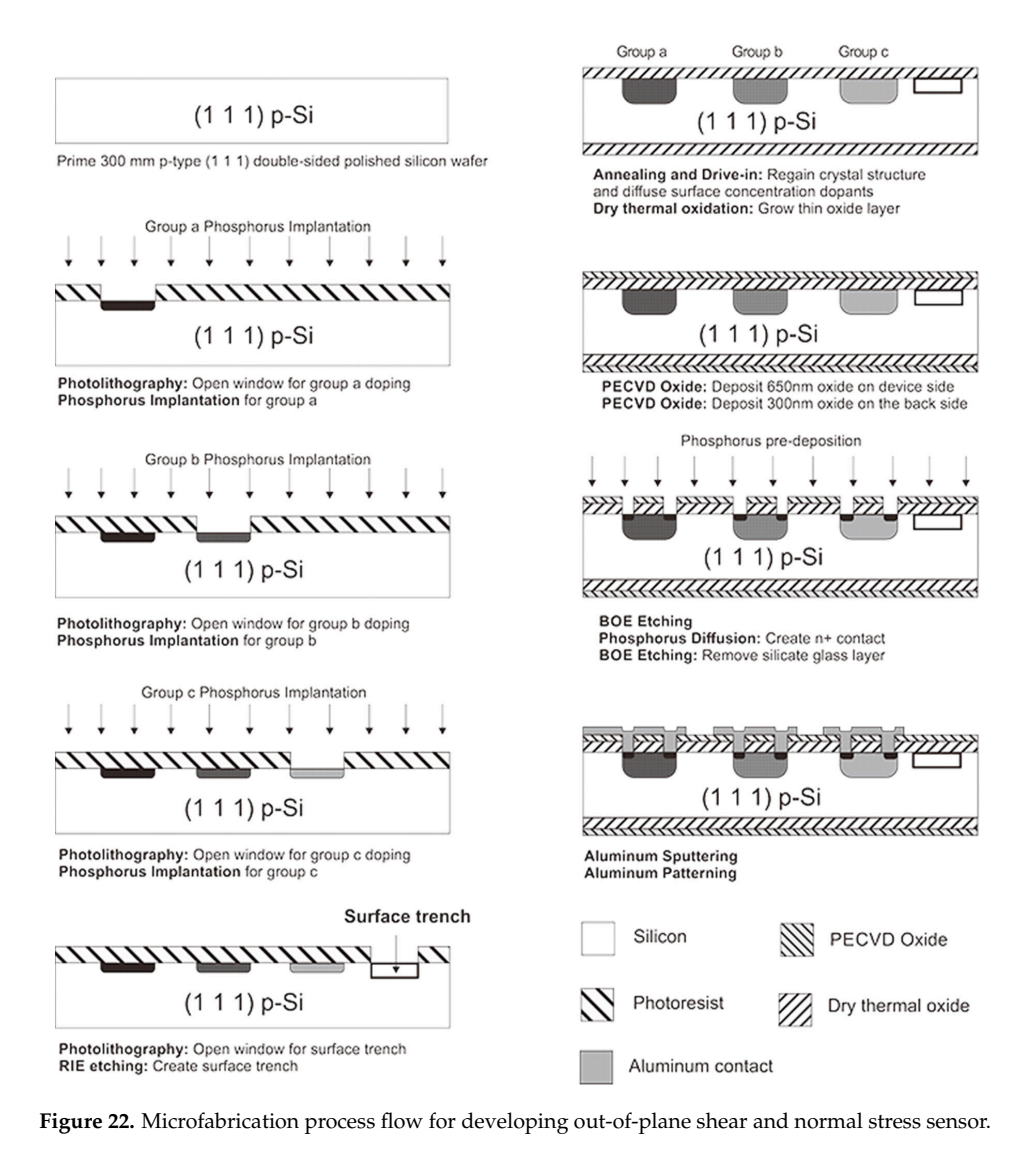
Microfabrication process flow for developing out-of-plane shear and normal stress sensor.

A microfabricaiton process flow is developed to prototype the proposed out-of-plane shear and normal stress sensor based on the analysis of geometric optimization, which is shown in Figure 22. The initial starting material was a p-type (111) single-sided polished prime silicon wafer with a diameter and thickness of 100 mm and 300 ± 25 µm respectively. The wafer is boron doped with bulk resistivity of 7 Ω-cm, which corresponds to a background impurity concentration of 2 × 10^15^ cm^−3^, followed by the ion implantation of groups a, b and c piezoresistors. The annealing and drive-in step was followed at 950 °C for 35 min, which included a 20 min annealing in an N2 atmosphere followed by 15 min dry thermal oxidation period. A layer of silicon dioxide (SiO_2_) using Plasma Enhanced Chemical Vapor Deposition (PECVD) is deposited for electrical insulation and as a masking layer for the next diffusion step. Etching of contact vias and n+ layer doping was conducted, followed by metallization.

**Figure 23:**
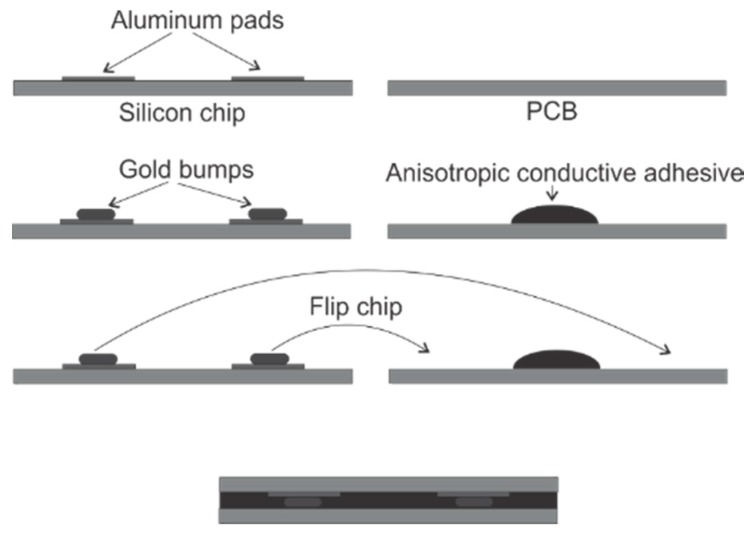
Proposed packaging scheme.

Packaging is a critical process for developing a MEMS out-of-plane shear and normal stress sensor. Flip chipping technique is adopted in our proposed packaging scheme, which is shown in Figure 23. The chip is bonded to the PCB beam using an anisotropic conductive adhesive (ACA), which is made up of conductive micron-sized particles floating in an epoxy resin matrix. This is followed by an applied normal pressure and temperature to cure the adhesive and create electrical conduction between the conductive particles and the pads on the chip and PCB. A wire-bonder was used to bond a number of gold (Au) stud bumps on the chip’s aluminum pads. On each pad, 5 stud bumps were bonded to cover the 350 × 350 mm^2^ pad area in order to provide large surface area for conduction. After packaging, signal generated from the sensor chip can be received using Oscilloscope connected to the PCB.

**Figure 24:**
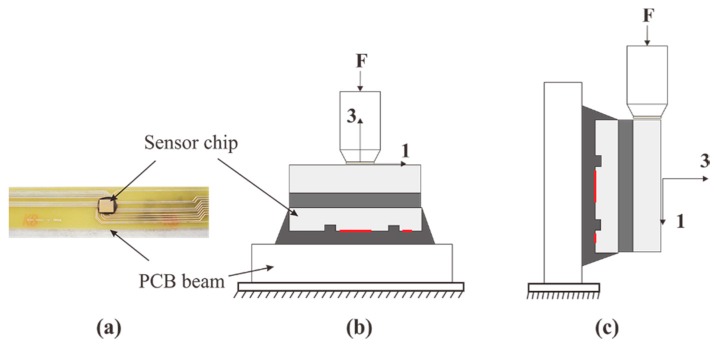
Assembled MEMS stress sensor on PCB beam (**a**) and schematic description of test setup for the measurement of out-of-plane normal stress $\sigma_{33}$ (**b**) and out-of-plane shear stress $\sigma_{13}$ (**c**).

A preliminary experimental testing has been conducted to verify the capacity of the proposed out-of-plane shear and normal stress sensor in extraction of the out-of-plane shear stresses and normal stress. The sensor chip with surface trenches are flip-chipped on a PCB following the procedure presented in Figure 23. The assembled sensor system was tested using a MTS universal testing machine, as shown in Figure 24. 

**Figure 25:**
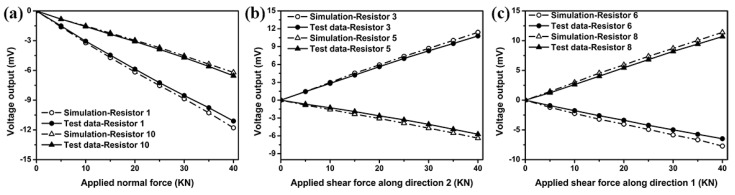
Proposed packaging scheme.

Out-of-plane shear force and normal force are applied to the stress transmission element individually. Voltage output from each resistor located in three sensing areas, i.e., center sensing area, edge sensing areas 1 and 2 are experimentally measured and compared with those determined from the FEM. Figure 25a–c compare the voltage outputs between FE simulation and experimental tests from piezoresistors 1 and 10 located in center sensing area, and from piezoresistors 3 and 5 located in edge sensing area 1, and from piezoresistors 6 and 8 located in edge sensing area 2, respectively. The results in Figure 25 indicate the currently simulation agree very well with the experimental values. 

**Figure 26:**
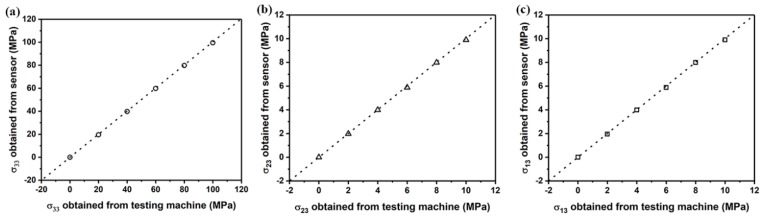
Comparison of out-of-plane normal stress $\sigma_{33}$ (**a**) and shear stresses $\sigma_{23}$ (**b**) and $\sigma_{13}$ (**c**) measured from the sensor chip and the load cell of universal testing machine.

In addition, the out-of-plane normal stress and shear stresses obtained from the proposed sensor in this paper were compared with those measured from the load cell installed in the testing machine. Results in Figure 26 show good agreement between the measured out-of-plane normal and shear stresses measured using the proposed sensor and the load cell installed in the testing machine.

**Figure 27:**
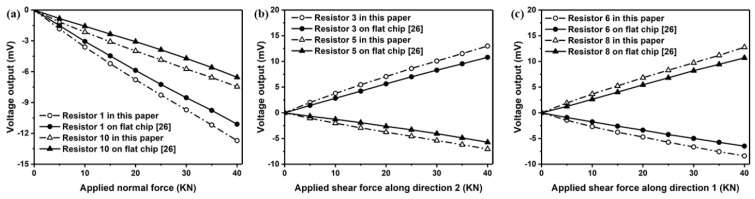
Comparison of signal output from the proposed sensor and the sensor in [22] for measuring out-of-plane normal stress $\sigma_{33}$ (**a**) and shear stresses $\sigma_{23}$ (**b**) and $\sigma_{13}$ (**c**).

The sensor performance of the proposed sensor chip with surface trenches has been compared with flat sensor chip reported in [22]. Results presented in Figure 27 suggest that the signal output has been improved by introducing surface trenches on the sensor chip.

## 7. Conclusions

A new methodology to realize MEMS piezoresistive out-of-plane shear and normal stress sensors was presented. This methodology emphasized the feasibility of utilizing stress concentration regions (SCRs), namely surface trenches as a mean to improve the sensor performance. A finite element model (FEM), verified by a five-layer analytical model was applied to guide the geometric optimization of the surface trenches. Results determined from FEM suggest that the sensor sensitivity can be improved by the incorporation of surface trenches to the vicinity of sensing areas. More specifically, the sum of percentage signal output from the sensor chip with surface trenches are 5.52, 6.06 and 6.72 times those from flat sensor chip for center sensing area, edge sensing areas 1 and 2, respectively. A microfabrication and packaging procedure were presented to develop the proposed out-of-plane shear and normal stress sensors with optimum geometric dimensions of surface trenches. Good agreement has been achieved between the FE simulation and experimental results. Detailed testing and calibration of the proposed out-of-plane shear and normal stress sensor will be presented in out next paper.

## Figures and Tables

**Table 1:** Pressure sensors with different structures and load ranges.

Sensor Type	Sensing Mechanism	Load Range	References
Diffused resistor Silicon diaphragm	Resistive	0–80 KPa	[27]
		20–200 KPa	[28]
		0–50 MPa	[29]
		0–100 KPa	[30]
Ion-implanted resistor Silicon diaphragm Silicon fusion bonding		103.4 KPa–34.5 MPa	[31]
Poly-silicon resistor Diaphragm		0–13.8 MPa	[32]
		0–137.9 KPa	[33]
Strain gauge Polymer diaphragm		0–10 KPa	[34]
Capacitor Diaphram	Capacitive	0–10 MPa	[35]
		0–178 KPa	[36]
		80–106 KPa	[37]
Resonator Diaphram	Resonant	0–550 KPa	[38]
FBGs Metal diaphragm	Optical	0–689.5 KPa	[39]

**Table 2:** Material properties and geometry of shear stress sensor system.

Components	Dimensions, mm	Material Properties
Silicon chip	7 × 7 × 0.3	C_11_ = 165.7 GPa C_12_ = 63.9 GPa C_44_ = 79.6 GPa
ACA	7 × 7 × 0.07	E = 3.3 GPa, ν = 0.3
PCB	180 × 22.73 × 1.57	E = 23.73 GPa, ν = 0.117
Gold Bumps	Φ 0.35 × 0.07	E = 77.2 GPa, ν = 0.3
Stress Transmission Element	7 × 7 × 2	E = 200 GPa, ν = 0.3

C_11_, C_12_, C_44_ = stiffness constants, E = Young’s modulus, ν = Poisson’s ratio.
